# The Long Issue in the Calvarium

**Published:** 1844-11-16

**Authors:** 


THE LONG ISSUE IN THE CALVARIUM.

In the Provincial Medical Journal is detailed by Dr. Oke, the case of a boy suffering from cerebral disease following scarlatina, in which advantage was derived from the long issue made in the calvarium, according to the directions of Dr. Wallis. Consciousness returned by the third day.—*Ibid.*

